# Understanding the Complexity of Early-Onset Dementia

**DOI:** 10.7759/cureus.57897

**Published:** 2024-04-09

**Authors:** Nicole Ann E Villa, Charles Wen, Eduardo D Espiridion

**Affiliations:** 1 Psychiatry, Drexel University College of Medicine, Philadelphia, USA; 2 Psychiatry, West Virginia School of Osteopathic Medicine, Lewisburg, USA; 3 Psychiatry, Philadelphia College of Osteopathic Medicine, Philadelphia, USA; 4 Psychiatry, Reading Hospital Tower Health Systems, West Reading, USA

**Keywords:** alzheimer’s disease, early-onset dementia, dementia, diagnostic evaluation, neurocognitive disorders

## Abstract

Dementia, particularly Alzheimer’s disease, affects millions globally, with its prevalence increasing notably with age. Early-onset Alzheimer’s disease, however, affects individuals under 65 years old. Unfortunately, diagnosing dementia in patients under 65 years old is quite challenging and is often delayed, missed, or wrong. Thus, we present the case of a 60-year-old female, with a medical history of hypothyroidism and presumed dementia on donepezil, who presented to the emergency department for agitation, dramatic change in personality and behavior, as well as cognitive decline that started in her late 50s. We discuss the importance of performing a thorough history and physical examination, as well as a comprehensive workup for patients who present with dramatic changes in behavior due to the wide range of potential diagnoses. While certain reversible causes, such as hypothyroidism, nutritional deficiencies, and polypharmacy, can be promptly identified and treated, chronic neurocognitive disorders such as Alzheimer’s disease demand a timely evaluation for early multidisciplinary treatment to enhance patient outcomes.

## Introduction

Dementia is a progressive disorder that involves the decline in function of at least one cognitive domain such as executive function, language, learning, memory, and social cognition. According to the World Health Organization 2023 dementia update, more than 55 million individuals were identified to have dementia worldwide [1]. Alzheimer’s disease is one cause of dementia and has been identified as the most common one, with an estimated 6.7 million Americans ages 65 and older living with Alzheimer’s in 2023. The percentage of individuals with Alzheimer’s dementia increases with age, where it is diagnosed in 5% of people ages 65 to 74 compared to 33.3% of people ages 85 and older. Studies have shown that individuals younger than 65 can develop Alzheimer’s which is referred to as early-onset Alzheimer’s disease (EOAD) [2].

Data on the prevalence and incidence rate of EOAD in the United States are challenging to determine given limited research. A few studies have estimated that EOAD makes up about one-third of individuals with dementia diagnosed before 65 years of age, with the remaining due to causes such as frontotemporal dementia (FTD), Lewy body dementia, and autoimmune or infectious conditions [3]. EOAD was shown to have an annual prevalence rate of 24.2 per 100,000 and an annual incidence rate of 6.3 per 100,000 for individuals between 45 and 64 years old [4,5]. Unfortunately, the diagnosis of dementia in patients below 65 years old is challenging to diagnose and is often missed where there is an average delay of 1.6 years for its diagnosis in comparison to older patients [6]. Thus, we present the case of a 60-year-old female patient who presented to the emergency department (ED) for cognitive decline, agitation, and dramatic change in personality and behavior.

## Case presentation

A 60-year-old female with a medical history of hypothyroidism and presumed dementia presented to the ED with cognitive decline, agitation, and dramatic change in personality and behavior. One week before admission, the patient had used public transport to visit her family but missed the bus back home and was lost wandering around for hours. On the day before admission, the husband noted that the patient woke up agitated overnight and was yelling and screaming. The family was unable to console the patient, leading the husband to call Emergency Medical Services (EMS) to bring her to the ED. According to EMS, the patient was erratic and aggressive, requiring rapid administration of 2 mg of Ativan for safe transport.

**Figure 1:**
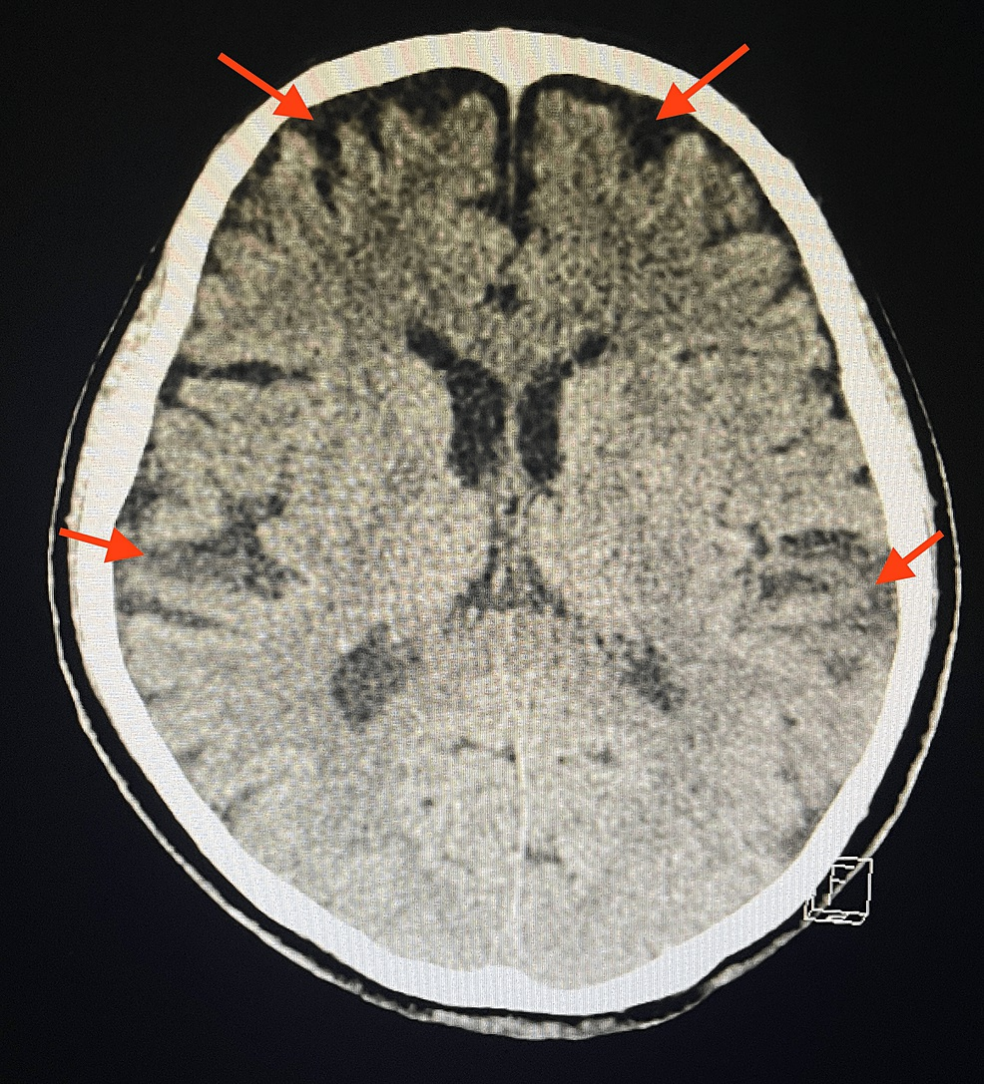
Non-contrast CT brain imaging performed in the emergency department. Non-contrast CT brain imaging showed that the ventricles, sulci, and cisterns were mildly prominent due to diffuse mild atrophy (red arrows). No midline shift, mass effect, mass lesion, hemorrhage, extra-axial collections, and acute infarction or fluid collections were seen.

On examination in the ED, the patient appeared lethargic, confused, and tired. She was only oriented to self, not to time and place. The neurological examination was extremely limited due to confusion and the patient was unable to follow basic commands. In addition, the examination was challenging as the husband noted that she was a non-native English speaker and could only understand some simple conversations. Vitals on arrival were significant for hypertension with a blood pressure of 155/109 mmHg. Laboratory results on admission were significant for severe hypokalemia of 2.8 mmol/L which was low (Table 1). Non-contrast CT of the brain showed that the ventricles, sulci, and cisterns were mildly prominent due to mild atrophy with no acute intracranial abnormalities (Figure 1). EKG showed no evidence of abnormal intervals or acute ischemic changes. Urinalysis was negative for urinary tract infection with no leukocyte esterase and nitrites detected. Serum and urine toxicology screens were negative for alcohol, salicylate, acetaminophen, and any recreational drugs.

**Table 1:** Laboratory tests ordered in the emergency department. Laboratory results were significant for severe hypokalemia.

Laboratory component	Results	Normal range
Sodium	144 mmol/L	136–145 mmol/L
Potassium	2.8 mmol/L	3.5–5.1 mmol/L
Creatinine	0.72 mg/dL	0.6–1.3 mg/dL
Alkaline phosphatase	53 IU/L	34–104 IU/L
Albumin	4.0 g/dL	3.5–5.7 g/dL
Total protein	6.5 g/dL	6.4–8.9 g/dL
Aspartate transaminase	18 IU/L	13–39 IU/L
Alanine transaminase	8 IU/L	7–52 IU/L
Total bilirubin	0.7 mg/dL	0.3–1.0 mg/dL
Direct bilirubin	0.1 mg/dL	0.0–0.2 mg/dL
White blood cell	4.4 × 10^3^/µL	4.8–10.8 × 10^3^/µL
Red blood cell	4.01 × 10^6^/µL	4.0–5.4 × 10^6^/µL
Hemoglobin	12.4 g/dL	12.0–16.0 g/dL
Hematocrit	35.7%	35.0–47.0%
Mean corpuscular volume	90.4 fL	80.0–99.0 fL
Platelets	168 × 10^3^/µL	130–400 × 10^3^/µL

According to her husband, the patient had been married for 13 years and retired from being a massage therapist. The patient was previously doing well and was independent with activities of daily life until about four years before admission when the husband started noticing changes in her behavior. He noted that she was becoming more forgetful and paranoid and had been having visual hallucinations. She was becoming increasingly isolated and socially withdrawn, with little desire to leave the home to see family or friends. She had frequent episodes of searching for objects that she had misplaced and accused others of stealing these items. In addition, she was often suspicious of others due to thoughts that her food was being laced with poison. The episodes occurred randomly at any time of day but are most frequently noted at night. These typically last a few hours, and she would suddenly return to her baseline with no memory of these episodes. The husband tried to take the patient to the emergency room multiple times when these episodes took place, but each time they arrived, her behavior was back to baseline.

She had been following up with her primary physician and neurologist since these issues first started, where she was diagnosed with hypothyroidism and possible EOAD. She was prescribed levothyroxine and memantine but had been non-compliant with these medications for a few months. The husband noted that the patient’s family lived in another country, with a family history significant for dementia in the patient’s mother and father.

Neurology and psychiatry were consulted on admission. During their examination, the patient was alert and oriented to self, time, and place. Cranial nerves II to XII were intact and within normal limits. Motor tone, strength, light touch, and temperature sensation were within normal limits as well. Reflexes were significant for +1 Achilles reflex bilaterally. No dysmetria for the finger-to-nose test and absent Romberg were noted. The Montreal Cognitive Assessment test was administered with a score of 12. This was administered with a language interpreter.

**Figure 2:**
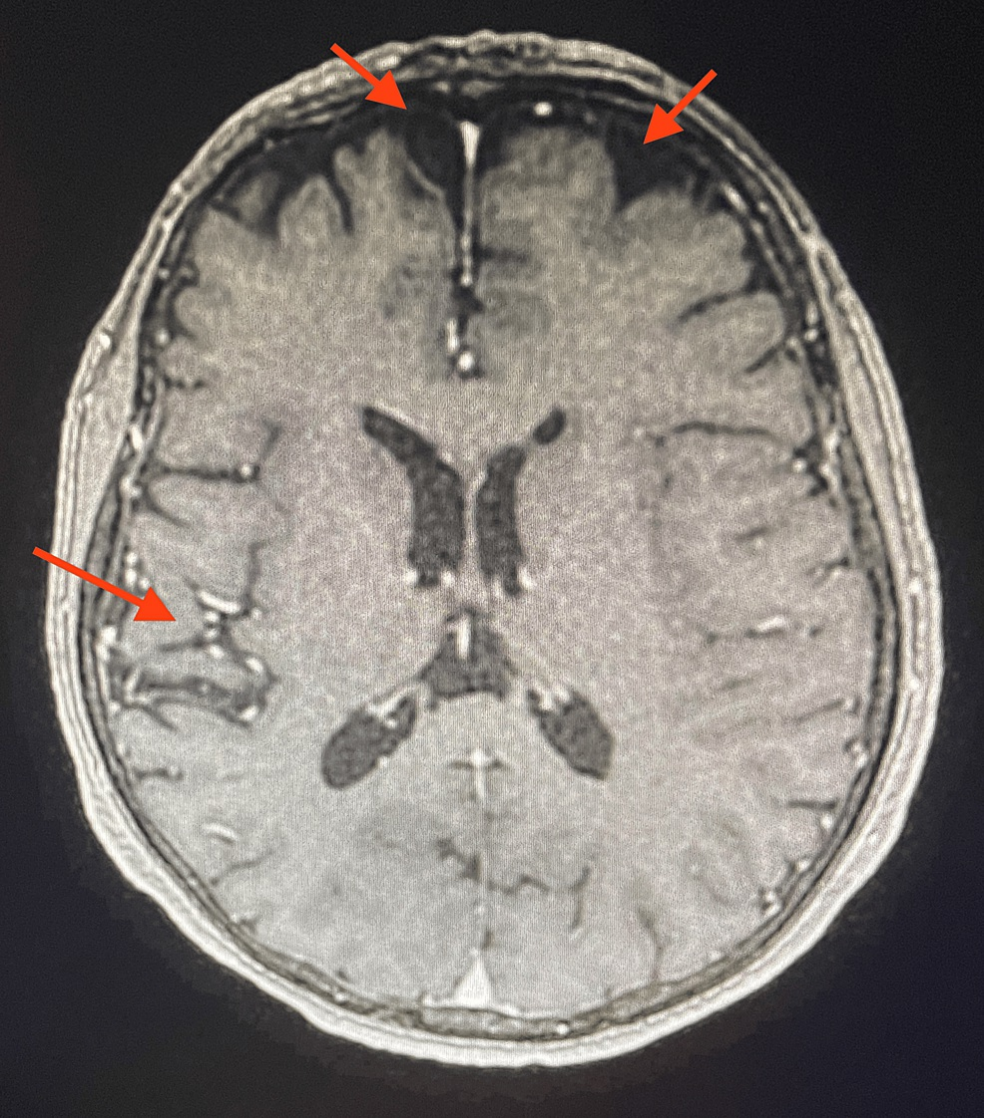
MRI with and without contrast ordered during admission. MRI showed evidence of mild frontotemporal atrophy and microangiopathy (red arrows). No acute infarcts, masses, or mass effects were observed.

During admission, the patient had multiple episodes of confusion and agitation, for which she was given risperidone 0.25 mg as needed. Further workup was performed at this time where thyroid-stimulating hormone (TSH), vitamin B12, methylmalonic acid, and vitamin B1 (thiamine) were ordered (Table 2). Vitamin B12 and B1 levels were low and appropriately corrected. In addition, an MRI with and without contrast was ordered to examine structural abnormalities where imaging found evidence of mild frontal and temporal atrophy and microangiopathy (Figure 2). Electroencephalography was also used to assist in staging encephalopathy where it showed generalized background slowing indicative of diffuse cerebral dysfunction, as seen in toxic metabolic and multifocal brain abnormalities. In addition, intermittent polymorphic delta slowing was seen independently in the right more than the left temporal regions, suggesting focal cerebral dysfunction.

**Table 2:** Laboratory tests ordered for workup during admission. Laboratory results were significant for low vitamin B12 and B1 levels.

Laboratory component	Results	Normal range
Folic acid	11.7 ng/mL	>5.9 ng/mL
Vitamin B12	177 pg/mL	180–914 pg/mL
Methylmalonic acid	109 nmol/L	87–318 nmol/L
Intrinsic factor-blocking antibody	Negative	Negative
Vitamin B1 (thiamine)	69 nmol/L	78–185 nmol/L
Thyroid-stimulating hormone	3.106 µIU/mL	0.45–5.33 µIU/mL

Given the results of the imaging, neurology had a primary working diagnosis of FTD; however, it was recommended that she undergo further outpatient workup with neuropsychology evaluation as well as tertiary care evaluation for neurocognitive and neurodegenerative disorders. At discharge, the patient showed overall improvement with her mentation close to baseline and was prescribed thiamine, vitamin B12, and memantine. During follow-up with her outpatient neurologist, she underwent nuclear medicine positron emission tomography brain imaging. Results found asymmetrically decreased cortical radiopharmaceutical fluorodeoxyglucose contrast uptake corresponding to the temporoparietal regions, suggestive of Alzheimer’s-type dementia. The patient was then started on donepezil 5 mg daily for dementia.

## Discussion

Patients who present with insidious forgetfulness and paranoia require an extensive workup as the range of potential diagnoses is extensive. Some causes such as hypothyroidism, nutritional deficiencies, polypharmacy, and substance abuse can be identified and treated relatively quickly. However, without these conditions, chronic neurocognitive disorders must be promptly evaluated as the early initiation of multidisciplinary treatment can improve patient outcomes.

Major neurocognitive disorder

Major neurocognitive disorder, also known as dementia, is a decline in cognition that is severe enough to cause significant disruption of independent daily function. It is best characterized as a syndrome with a range of etiologies, including Alzheimer’s disease, vascular disease, frontotemporal lobar degeneration, Lewy body disease, Parkinson’s disease, HIV infection, Huntington’s disease, prion disease, substance and/or medication use, and traumatic brain injury. It is prevalent in 7% of individuals above the age of 65 years, with a slightly higher prevalence of 8-10% in developed countries due to longer life spans [7].

Early-onset Alzheimer’s disease

Alzheimer’s disease is the most common cause of dementia in patients over and under 65. Alzheimer’s disease occurring in patients under 65 is classified as EOAD. It occurs in 4-6% of all Alzheimer’s disease and is the most common early-onset neurodegenerative dementia [8]. It is important to recognize that EOAD is not merely an epidemiological extension of late-onset Alzheimer’s disease (LOAD). Patients with EOAD often present with a more aggressive clinical course, and when compared to comparably impaired LOAD patients, have better memory recognition scores and semantic memory but worse attention, executive functions, ideomotor praxis, and visuospatial skills. Furthermore, 22- 64% of patients with EOAD present with non-amnestic, variant phenotypes, known as type 2 Alzheimer’s disease and include logopenic variant primary progressive aphasia, posterior cortical atrophy, progressive ideomotor apraxia, and behavioral/dysexecutive variants. The early onset and variability of symptoms often contribute to delays in diagnosis and treatment [9].

Frontotemporal dementia

FTD is the second most common cause of dementia in patients under the age of 65. It is a cluster of neurocognitive disorders defined by progressive dysfunction in behavior, language, and function. The underlying pathological mechanism is characterized by intracellular deposition of abnormal protein aggregates in the frontal and temporal lobes. Diagnosis is made clinically but is challenging as the initial presentation can appear as a psychiatric disorder or stroke. A thorough history and physical examination, as well as collateral information from caretakers, is critical; however, laboratory tests, neuroimaging, and neurocognitive testing can aid in diagnosis. The clinical presentation of FTD can be broadly divided into three subtypes, namely, the behavior variant, semantic variant, and non-fluent variant, primary progressive aphasia [10].

Pseudodementia

Pseudodementia describes the cognitive deficits that resemble dementia but are caused by other conditions, most often depression. It is characterized by deficits in memory, executive function, and speech and is most often seen in older adults. Pseudodementia is widely considered a reversible cause of dementia and management is aimed at treating the underlying depressive disorder. However, an observational study found that more than 70% of patients presenting with pseudodementia converted to overt dementia, suggesting that pseudodementia is a predictor of developing dementia later [11].

Thyroid issues

Hypothyroidism results from low levels of thyroid hormone and can be caused by a range of etiologies. Iodine deficiency is the most common cause of hypothyroidism globally; however, in the United States, autoimmune thyroid disease is the most common cause of hypothyroidism. The thyroid gland plays a crucial role in regulating the body’s metabolism, including the brain’s metabolism and the function of neurotransmitters. Clinically, hypothyroidism can be broadly subdivided into overt hypothyroidism, defined as an elevated TSH and low T4, and subclinical hypothyroidism, defined by elevated TSH and normal T4. Subclinical hypothyroidism has been found to have no association with cognitive function, cognitive decline, or dementia [12]. However, the association between overt hypothyroidism, cognition, and mood is well-established [13].

Overt hypothyroidism commonly causes slowing of thought, apathy, and decreased attentiveness. In 5-15% of cases, overt hypothyroidism can present as psychosis and is sometimes referred to as “myxedema madness.” Hypothyroidism is one of the reversible causes of dementia and psychosis; treatment with replacement T4 results in the gradual resolution of cognitive and psychiatric symptoms [14].

Vitamin B12 deficiency

Vitamin B12, also known as cobalamin, is a water-soluble vitamin derived from animal products such as red meat, dairy, and eggs. Vitamin B12 is an important cofactor for enzymes in two important metabolic reactions. As a cofactor for methionine synthase, vitamin B12 is used in the conversion of homocysteine to methionine which generates tetrahydrofolate (THF) from methyl-THF. THF is converted to intermediates in the synthesis of pyrimidines used in DNA synthesis. Vitamin B12 deficiency in this pathway leads to the classic megaloblastic anemia associated with vitamin B12 and folate deficiency. Vitamin B12 is also a cofactor for the enzyme methylmalonyl-CoA mutase, which converts methylmalonyl-CoA to succinyl-CoA. When vitamin B12 is deficient, methylmalonyl-CoA accumulates. Elevated levels of methylmalonyl and homocysteine are believed to cause damage to myelin, which, if left untreated, can lead to a subacute combined degeneration a condition of the spinal cord characterized by loss of proprioception, ataxia, peripheral neuropathy, and dementia. Vitamin B12 deficiency represents a reversible cause of dementia, as 86% of patients will have resolution of symptoms after treatment [15].

In some cases, psychiatric symptoms have been documented as the presenting symptom of vitamin B12 deficiency. A series of middle-aged vegetarians presenting with psychosis refractory to anti-psychotic medication were found to be mildly deficient in vitamin B12 without major neurological or hematological deficits [16].

Substance abuse

Substance abuse, especially with drugs and alcohol, may have detrimental effects on the brain and can lead to cognitive impairments and behavioral changes. Tetrahydrocannabinol found in cannabis and psychedelic drugs such as lysergic acid diethylamide, psilocybin, N, N-dimethyltryptamine, and mescaline can cause behavior changes, hallucinations, and delusions that may present as psychosis. Alcohol intoxication can cause acute mental status changes that can be mistaken for psychiatric symptoms. Amphetamine use can also present with agitation and in some cases psychosis.

Additionally, substances of abuse, particularly alcohol, have been found to be neurotoxic and can induce structural and functional changes in the brain. These changes manifest in memory impairment, cognitive decline, motor impairment, and hallucinations and delusions. While mental status changes due to intoxication are often acute and self-resolving, cognitive and behavioral changes due to chronic substance abuse are often irreversible [17].

This patient with intermittent changes in personality and behavior beginning in her late 50s most likely has EAOD, potentially a behavior or dysexecutive variant. EAOD is associated with delays in diagnosis, distress and confusion over symptoms, and profound psychological distress [8]. However, a range of other diagnoses should also be considered. Her symptomology and imaging results might be interpreted by some as FTD. Furthermore, her symptomatic improvement with administration of vitamin B12 and folate may suggest a rare presentation of vitamin B12 deficiency. The patient’s early intermittent memory impairments, behavioral changes, and social isolation could be attributed to an underlying depressive disorder. Lastly, overt hypothyroidism due to the patient’s history of medical non-compliance and underlying thyroid disease could explain her symptoms but are unlikely due to her normal thyroid function at presentation.

Furthermore, it is important to note that diagnosis can be particularly challenging in patients who are non-native English speakers from culturally diverse backgrounds such as the patient described. A longitudinal study found a two-to-three-fold increase in the prevalence of mild cognitive impairment in participants with non-English-speaking backgrounds compared to participants with English-speaking backgrounds when using traditional neurocognitive testing methods [18]. Another study reviewed a range of existing neurocognitive testing methods and their potential efficacy in assessing patients of low-educational and non-western backgrounds. Tests that incorporated clear, colored images and did not require pencil and paper, such as the colors trails test and five-digit test, were found preferable to tests typically found on traditional assessment batteries such as the trail-making test and clock drawing test [19]. While no testing methodology was found to be definitively superior in assessing patients of non-western backgrounds, the review highlighted the need for further study given an increasingly diverse patient population in the United States.

## Conclusions

The range of potential etiologies for this patient’s change in behavior and personality is broad, highlighting the importance of a careful and comprehensive workup in this group of patients. History and physical examination should be the clinicians’ first tools with special attention paid to discerning the age of onset and symptomology. Although epidemiological studies are limited, current data suggests that Alzheimer’s dementia is the most common cause of dementia in patients under the age of 65. Clinicians should be aware that EOAD presents with atypical symptoms and a more progressive disease course. Laboratory testing should evaluate thyroid function, nutritional deficiencies, and potential intoxication. Imaging can be useful in diagnosis once all major etiologies have been excluded but is not necessary. Furthermore, clinicians must be mindful of their patient’s linguistic proficiency and cultural backgrounds and how these factors might impact diagnosis and management. This is particularly important in this case where language interpreter services were utilized. Overall, the difficult nature of diagnosing EOAD has resulted in a limited epidemiological understanding of this disease. EOAD may be more prevalent than previously thought; regardless, clinicians should have a low index of suspicion for this condition when assessing cognitive and behavioral changes in patients under 65.
